# Assessment of the stresses produced on the bone implant/tissue interface to the different insertion angulations of the implant - a three-dimensional analysis by the finite elements method

**DOI:** 10.4317/jced.57387

**Published:** 2020-10-01

**Authors:** Joelson-Rodrigues Brum, Fabiano-Rito Macedo, Millena-Barroso Oliveira, Luiz-Renato Paranhos, Rui-Barbosa Brito-Júnior, Juliana-Cama Ramacciato

**Affiliations:** 1 DDs, MSc, PhD student, Faculdade São Leopoldo Mandic, Campinas, SP, Brazil; 2 DDs, Post-Graduation Program in Dentistry, Federal University of Uberlândia (UFU), Uberlândia, MG, Brazil; 3 DDs, MSc, PhD, Department of Community and Preventive Dentistry, Federal University of Uberlândia (UFU), Uberlândia, MG, Brazil; 4 DDs, MSc, PhD, Faculdade São Leopoldo Mandic, Campinas, SP, Brazil

## Abstract

**Background:**

The present study aimed to assess the stresses produced on the surface of the bone tissue around dental implants with three different insertion angulations subjected to axial and oblique loading.

**Material and Methods:**

The study was created according to the recommendations of the Checklist for Reporting In-vitro Studies (CRIS). The Straumann™ bone level RC (4.1 x 10 mm) implant, Cone Morse connection (CM), RC Straumann Variobase™ with abutment (3.5 mm) was placed in the region of element 16, with the platform positioned at the height of the bone crest. Three assessment models were produced: model M1 or control - implant perpendicular to the bone crest; model M2 - implant angulated at 17° relative to the bone crest; and model M3 - implant angulated at 30° relative to the bone crest. The masticatory loads were simulated with 100 N of intensity and two loading patterns (axial and oblique) were applied to each model. Then, the models were exported to the finite elements simulation software Ansys Workbench V19.2 (Ansys Inc., Canonsburg, PA, USA). To assess the finite elements, qualitative and quantitative analyses were performed.

**Results:**

It was observed that, under axial loading, qualitatively, the peaks occurred in the cavosurface region, palatal aspect in M1 and M2, and buccal aspect in M3. Quantitatively, the greatest angulation resulted in a low stress peak. Under oblique loading, qualitatively, the peaks occurred in the cavosurface region, buccal aspect in the three groups. Quantitatively, the greatest angulation of the implant resulted in an increase in stress peaks on the buccal aspect.

**Conclusions:**

Under axial loading, the three insertion angulations of the implant - M1, M2, and M3 - were clinically viable. When subjected to oblique loading, the 30° angulation (M3) suggested a significant risk of bone loss and it was contraindicated.

** Key words:**Finite element analysis, dental implants, load support.

## Introduction

Implantology has been focusing studies in the search for technological and minimally invasive advancements both in the surgical phase (1-3) and the prosthetic and component selection phase (4). The analyses of the mechanical performance of implant-supported prostheses involve studies aiming not only to assess the resistance of the implant-prosthetic crown set (5) but also to understand the bone behavior (5,6) to the potential variations of length, angulation, and implant positioning (7).

Thus, investigations on design modifications, surface treatments, and screw spacing are performed daily (8-10) to prevent bone loss, consequently increasing the rate of success with dental implant treatments (8).

Ideally, implants for dental prostheses should be positioned parallel to each other but certain clinical circumstances require angulated implants (11), so understanding the bone response to different load applications becomes essential. Thus, the present study aimed to perform a three-dimensional analysis of the stresses produced on the surface of bone tissue around implants when subjected to axial and oblique loading. The null hypothesis tested in this study was that different insertion angulations of the implant would not affect the stress produced on the adjacent bone tissue.

## Material and Methods

The local ethics committee approved the study (protocol no. 2020-0097). The entire study was created according to the recommendations of the Checklist for Reporting *In-vitro* Studies (CRIS) (12).

-Obtaining the geometric models

The three-dimensional model of the dentate and edentulous maxilla came from a pre-existing model available in the literature for the use of the scientific community (13). The models were downloaded in the parametric format using the SolidWorks 2011™ software. The geometric changes required in the model were performed in the CAD Solidworks 2017 software (Dassault Systemes, Solidworks Corps, USA).

To obtain the geometric models of the implant and the components used in the study, they were subjected to reverse engineering with a digital caliper (Mod. 500-196-30B, Mitutoyo Sul Americana Ltda., Suzano, Brazil), digital microscope (MV500UM-PL, Cosview Technologies Co. Ltd, Bantian, China) with a magnification of 5x~200x, and a measuring software (Miviewcap 6.0, Cosview Technologies Co. Ltd, Bantian, China) to measure the geometry of the components and allow molding in the Solidworks software.

-Sample preparations

**Figure 1:**
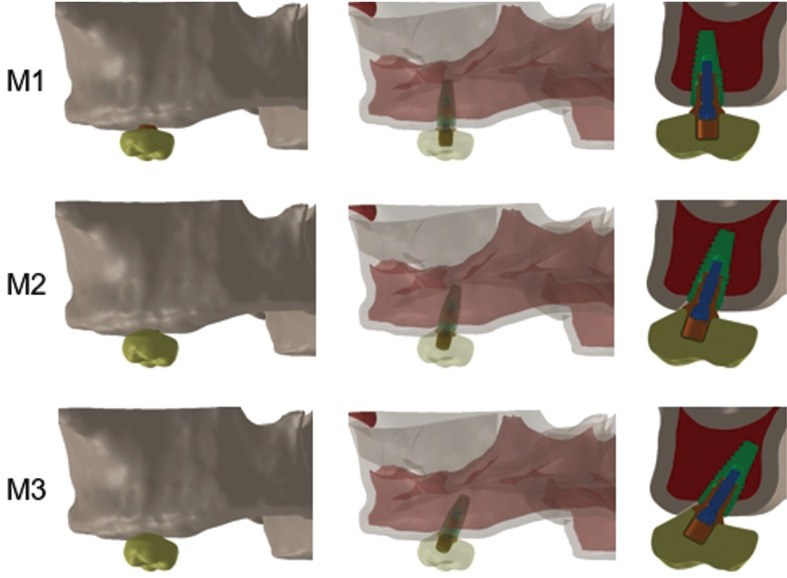
Models analyzed with different angulations relative to the bone crest. M1 (perpendicular); M2 (17°); M3 (30°).

The Straumann™ bone level RC (4.1 x 10 mm, Institut Straumann AG, Basel, Switzerland) implant, Cone Morse connection (CM), with RC Variobase™ abutment (3.5 mm, Institut Straumann AG, Basel, Switzerland) was placed in the region of element 16 with the platform positioned at the height of the bone crest, and it was considered osseointegrated. The position of the implant presented a variable angulation according to the model (Fig. 1): M1 or control - with the implant perpendicular to the bone crest; M2 - with the implant angulated at 17° relative to the bone crest and apical portion facing the palate; and M3 - with the implant angulated at 30° relative to the bone crest and apical portion facing the palate.

The crown (lithium disilicate glass-ceramic, thickness of 1.5 mm, IPS e.max press, Ivoclar, Vivadent, Schaan, Liechtenstein) was cemented with a layer of 62 µm of resin cement between the restorative material and the prosthetic intermediate.

-Determination of contact points

The enamel structure that simulated the occlusal third of antagonist teeth received three circular contact points with 1 mm of diameter each, for axial loading application. One point was positioned in the buccal cusp and two other points in the palatal cusp, buccal and palatal sides. In turn, for applying the oblique loading, the points were positioned in the buccal sides of the lingual cusps. A bolus was added with the Young modulus similar to the almond, 21.57 (4.00) MPa (14) and a thickness of 2 mm, between the ceramic crown and the antagonist structure of the enamel, as a way to represent a condition as naturally as possible.

-Load application

The axial loading was applied with a parallel vector along the axis of the element, over the upper portion of the structure that simulated the antagonist teeth. In the occlusal contact, the antagonist structure was set with frictionless supports on the sides to allow a uniquely gingival occlusal movement. The contacts between the antagonist structure and the crowns were set as “frictionless”, allowing sliding and gap formation. This enabled both the intrusion and a buccolingual movement of the implants, similar to real-life conditions.

The oblique loading was simulated with a vector in the palatal-buccal direction, forming a 30° angle with the occlusal plane. The antagonist structure was used to standardize the loading area.

Rigid supports were added in the areas in which the maxilla would connect to the rest of the skull. The simulations were non-linear relative to the contact.

-Finite element analysis

All models were exported to the finite elements simulation software Ansys Workbench V19.2 (Ansys Inc., Canonsburg, PA, USA) using an import accessory of the software itself.

To represent the mechanical behavior of each component as reliably as possible, the different elements of the models were set based on the elasticity modulus and Poisson’s coefficient. All the material was considered linear, isotropic, and homogeneous. The implant (Roxolid™) required the mean between the elasticity modulus of a titanium alloy with 10% zirconia and 90% titanium and another with 20% zirconia and 80% titanium (15).

Non-linear frictional contacts with a friction coefficient of 0.2 µ (16) were used to simulate the contact between titanium surfaces. All the others were simulated with contacts that did not allow sliding or gap formation, except for the contact between the antagonist axial structure and the crown.

The masticatory loads were simulated with 100 N of intensity in the axial and oblique loading patterns.

The meshes of finite elements were created with a mesh refinement process until the variation reached 5% or less, indicating that the distortion by the mesh intensity would not affect significantly the results. The mesh was produced with quadratic tetrahedral elements of 10 knots (solid 187), which allowed copying the irregular geometry of the models analyzed. The number of knots/elements ranged from 1933932/1240795 to 1935240/1240714. All models were then resolved (Windows 10 64 bits, Intel I7 6800k processor, 112 Gb RAM) and the graphic and numerical plots of the data were assessed and compared.

To assess the finite elements, qualitative and quantitative analyses were performed.

-Structural quantification of the risk of peri-implant bone damage

**Figure 2:**
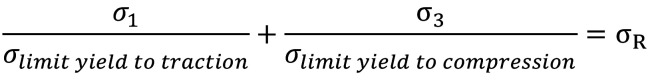
Formula.

The Mohr Coulomb criterion was used to quantify at a structural level the risk of peri-implant bone damage. The Mohr Coulomb theory defines that material will fracture when the combination of the main stresses are equal or exceed the resistance limits. The impact of tensile stresses and its relationship with tensile strength were analyzed, as well as the compressive stresses and their relationship with compressive strength. To facilitate the comparative analyses, an adaptation was performed and defined by the formula: (Fig. 2).

where σR is the result, σ1 is the main maximum stress, σ3 is the main minimum stress, and σlimit represents the maximum yield stress to compression and traction. As a reference for the calculation, the limit yield stress to traction was 82.8 MPa and the limit yield stress to compression was 133.6 MPa (17). Such values were based on the trabecular bone, considering the reference values were not found within the same study for the indexes of cortical bone. As a convenience, model M1 was defined as control.

## Results

**Figure 3:**
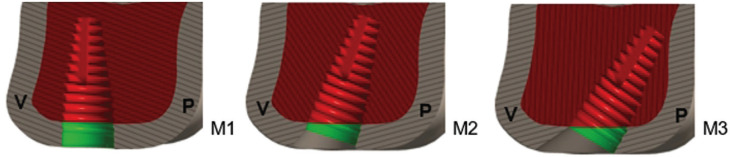
Peri-implant cortical bone insertion in the different models, highlighted in green. M1: perpendicular to the bone crest; M2: inclination of 17°; and M3: inclination of 30°.

Figure 3 shows a sectioned view of the peri-implant cortical insertions of models M1, M2, and M3.

**Figure 4:**
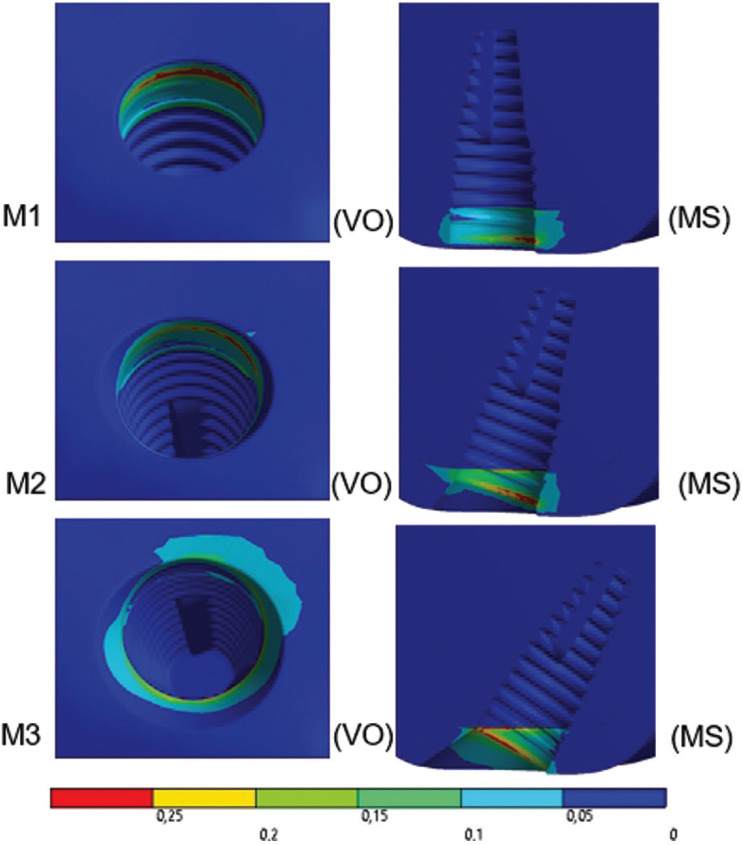
External sectioned view of the results in the peri-implant bone under axial loading. A linear color scale was used, where blue indicates low values and red indicates high values of stress on the peri-implant bone. Models: M1, M2, and M3. VO view: buccal occlusal; MS: sectioned mesial. Considering the view is sectioned, the mesial view shows the distal portion.

When analyzed qualitatively, the results of the peri-implant bone under axial loading (Fig. 4) showed that the peaks occurred in the cavosurface region, on the palatal side of models M1 and M2 and the buccal side of model M3. Quantitatively, it was observed that the greatest angulation resulted in low stress peaks and consequently risk of bone loss.

**Figure 5:**
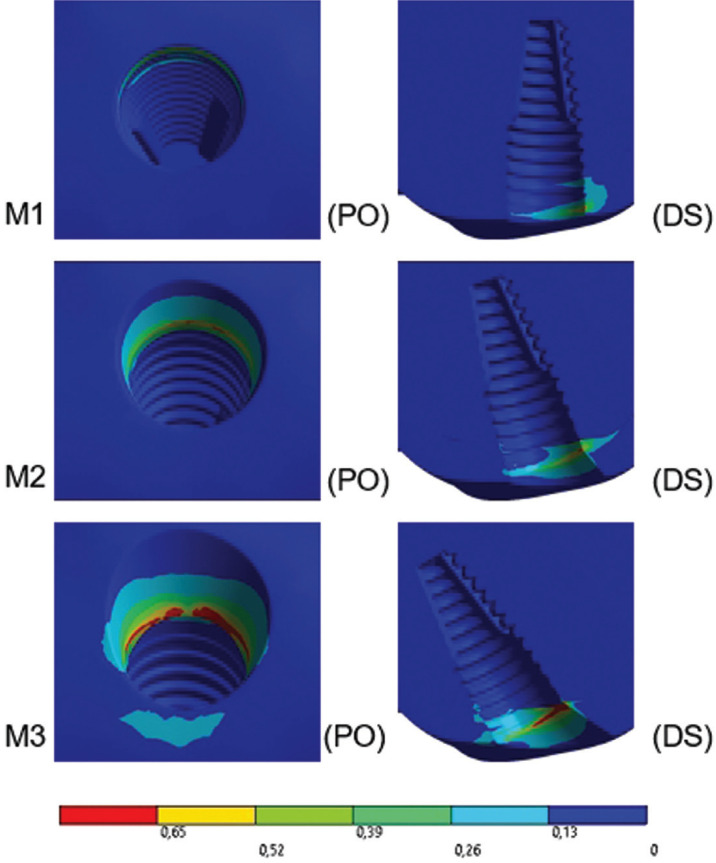
External sectioned view of the results in the peri-implant bone under oblique loading. A linear color scale was used, where blue indicates low values and red indicates high values of stress on the peri-implant bone. Models: M1, M2, and M3. PO view: palatal occlusal; DS: sectioned distal. Considering the view is sectioned, the distal view shows the mesial portion.

When analyzing the results of the peri-implant bone under oblique loading qualitatively (Fig. 5), the peaks occurred in the buccal cavosurface region in all models. Quantitatively, there was an inversion of the trends found for the axial loading, with the greatest angulation of the implant increasing stress peaks.

The peaks of the results in the peri-implant bone, according to the Mohr Coulomb Criterion and its percentage, to the axial loading were: M1 - 0.362 (100%); M2 – 0.351 (97%); M3 – 0.332 (91%). For the oblique loading, the results were: M1 – 0.741 (100%); M2 – 0.763 (103%); M3 – 1.208 (163%).

## Discussion

Three different insertion angulations of dental implants were simulated to assess the stress exerted on the adjacent bone when axial and oblique forces were applied. The hypothesis that different insertion angulations of the implant would not affect the stress produced on the adjacent bone tissue was rejected.

In the peri-implant bone under axial loading, qualitatively, the peaks occurred in the cavosurface region due to the closest proximity to the point of origin of the load in the cortical bone, affecting the palatal side in M1 (0°) and M2 (17°). In model M3 (30°), the positioning of the peak was buccal and it may be explained by the thinning of the cortical bone in the buccal aspect due to the degree of angulation. Winter *et al.* (18) observed that the stability of implants in the models studied was positively correlated to the length and thickness of the cortical bone, validating the results found. Thus, according to Kurniawan *et al.* (19), for minimum stress and tension in a certain region, a peri-implant bone that is denser and osseointegrated is desired. As for the oblique loading, when analyzing the results of the peri-implant bone qualitatively, the peaks occurred in the buccal cavosurface region in all models, especially because the horizontal component of the oblique force is buccally directed, without divergences in the region affected among the models.

Quantitatively, the axial loading showed that the greatest angulation (30°) resulted in low stress peaks and the risk of bone loss. In turn, Amid *et al.* (20) observed that the application of an axial loading at 100 N and 30° angle caused more stress when compared to the axial application of 300 N along the axis of implants, presenting different results from those found in the present study. For the oblique loading, quantitatively, there was an inversion of the trends found for the axial loading, in which the greatest angulation of the implant elevated the stress peaks. The data corroborate Amid *et al.* (20), who obtained maximum stress on the peri-implant bone when applying oblique loading at a 30° angle.

Oblique loading and implant inclination were also harmful in the study by Almeida *et al.* (21), which showed that when increasing implant angulation, the area of stress concentration also increased on the adjacent bone, especially under the application of oblique loading. In the same study, the distribution of compressive and tensile stresses presented values of lower intensity for the axial loading.

In the structural quantification of the risk of peri-implant bone damage, by the Mohr Coulomb method, the difference of peaks among the models was a maximum of 9% for the axial loading. In the oblique loading, the difference was 3% between models M1 (100%) and M2 (103%), suggesting a similar clinical performance accepTable between angulations. However, in model M3, the difference was 63% under oblique loading, showing a higher risk of bone loss with the increase of insertion angulation. In line with the findings, Hong *et al.* (7) observed in their study that as the implants were inclined, not only the levels of stress on the bone tissue increased but also the efficiency of stress distributions on the adjacent bone decreased. The authors also showed that the lowest stress and the best implant stability on mandibular overdentures were obtained when the implants were inserted in the regions of lateral incisors with shorter implants positioned parallel to the long axis of the teeth (7). Hence, M3 was contraindicated.

The implant model M1 presented the best results for stress on the peri-implant bone tissue when compared to models M2 and M3. This is mainly because the type and position of the implant affect directly the levels of stress produced, thus the implants placed along the loading axis show a better stress distribution (22). Watanabe *et al.* (23) verified that, regardless of the point and direction of the load application, the compressive stresses were relatively higher when the implant was inclined. Thus, when the load was applied with a direction of 45 degrees, the compressive stress on the cortical bone was adjacent to the implant inclination, as the elastic stress was restricted only to the opposite side.

For the clinical decision, the findings of the present study were rather significant because they showed that the relationship between insertion angulation of the implant and the load applied, in this case axial or oblique, affected directly the stress on the surrounding bone tissue. Studies show that the maximum equivalent stress increases linearly to the load angle increase, that is, for each increase of 30 degrees in the load angle, the maximum equivalent stress on the cortical bone increases in average 3 to 4 times in comparison to the axial loading applied (24).

As a limitation, the model of finite element analysis of this study could not simulate all the characteristics of the living tissue, although the simulation with bolus between the occlusal third of the ceramic crown and the antagonist structure of the enamel was able to affect which regions the peaks of axial and oblique loading applied would occur.

The finite element analysis allowed a satisfactory *in vitro* model simulation through the numerical/computer reproduction of the structures and materials involved in this study. Therefore, it is evident the significance of *in vitro* studies as a key point to a coherent clinical indication and predictability. This study showed that different insertion angulations, according to models M1, M2, and M3, caused different levels of stress on the underlying bone tissue when the models were subjected to axial and oblique loading. However, further clinical research should be performed to approximate the results found to the behavior of living tissues.

## Conclusions

The insertion angulations of the implant studied were clinically viable when applying axial loading. When subjected to oblique loading, the 30° angulation suggested a significant risk of bone loss and its clinical application should not be recommended.
